# COVID-19 vaccine type-dependent differences in immunogenicity and inflammatory response: BNT162b2 and ChAdOx1 nCoV-19

**DOI:** 10.3389/fimmu.2022.975363

**Published:** 2022-09-02

**Authors:** Jung Yeon Heo, Yu Bin Seo, Eun Jin Kim, Jacob Lee, Young Rong Kim, Jin Gu Yoon, Ji Yun Noh, Hee Jin Cheong, Woo Joo Kim, Soo-Young Yoon, Ju-Yeon Choi, Young Jae Lee, Hye Won Lee, Sung Soon Kim, Byoungguk Kim, Joon Young Song

**Affiliations:** ^1^ Department of Infectious Diseases, Ajou University School of Medicine, Suwon, South Korea; ^2^ Division of Infectious Disease, Department of Internal Medicine, Kangnam Sacred Heart Hospital, Hallym University College of Medicine, Seoul, South Korea; ^3^ Division of Infectious Diseases, Department of Internal Medicine, Korea University College of Medicine, Seoul, South Korea; ^4^ Asian Pacific Influenza Institute (APII), Korea University College of Medicine, Seoul, South Korea; ^5^ Vaccine Innovation Center-KU Medicine (VIC-K), Seoul, South Korea; ^6^ Department of Laboratory Medicine, Korea University College of Medicine, Seoul, South Korea; ^7^ Division of Vaccine Clinical Research, Center for Vaccine Research, National Institute of Infectious Diseases, Cheongju, South Korea

**Keywords:** COVID-19, vaccine, immunogenicity, reactogenicity, cytokine

## Abstract

Evaluation of the safety and immunogenicity of new vaccine platforms is needed to increase public acceptance of coronavirus disease 2019 (COVID-19) vaccines. Here, we evaluated the association between reactogenicity and immunogenicity in healthy adults following vaccination by analyzing blood samples before and after sequential two-dose vaccinations of BNT162b2 and ChAdOx1 nCoV-19. Outcomes included anti-S IgG antibody and neutralizing antibody responses, adverse events, and proinflammatory cytokine responses. A total of 59 and 57 participants vaccinated with BNT162b2 and ChAdOx1 nCoV-19, respectively, were enrolled. Systemic adverse events were more common after the first ChAdOx1 nCoV-19 dose than after the second. An opposite trend was observed in BNT162b2 recipients. Although the first ChAdOx1 nCoV-19 dose significantly elevated the median proinflammatory cytokine levels, the second dose did not, and neither did either dose of BNT162b2. Grades of systemic adverse events in ChAdOx1 nCoV-19 recipients were significantly associated with IL-6 and IL-1β levels. Anti-S IgG and neutralizing antibody titers resulting from the second BNT162b2 dose were significantly associated with fever. In conclusion, systemic adverse events resulting from the first ChAdOx1 nCoV-19 dose may be associated with proinflammatory cytokine responses rather than humoral immune responses. Febrile reactions after second BNT162b2 dose were positively correlated with vaccine-induced immune responses rather than with inflammatory responses.

## Introduction

The coronavirus disease 2019 (COVID-19) pandemic, caused by the severe acute respiratory syndrome coronavirus 2 (SARS-CoV-2), is an ongoing public health crisis since late 2019. Vaccination is the most effective strategy to minimize the devastating impact of the COVID-19 pandemic. Four different types of COVID-19 vaccines have been developed: the classical inactivated whole-virus vaccines, newly licensed messenger RNA (mRNA)-based vaccines, adenovirus-vector-based vaccines, and recombinant protein nanoparticle vaccines. Of these, BNT162b2 (mRNA vaccine developed by BioNTech/Pfizer) and ChAdOx1 nCoV-19 (chimpanzee adenovirus-vector vaccine developed by Oxford University/AstraZeneca), which were the initially approved vaccines, have been administered to a majority of the global population (1). These vaccine platforms have demonstrated favorable efficacy in clinical trials; BNT162b2 and ChAdOx1 nCoV-19 have shown 95% and 70.5% efficacy against COVID-19, respectively (2–3). Adverse events with mild-to-moderate severity have been reported in approximately 50–70% of the individuals vaccinated with either BNT162b2 or ChAdOx1 nCoV-19 (4). Approximately one-third of the vaccine recipients experience adverse reactions, which interfere with their daily activities (5).

Reactogenicity refers to the anticipated symptoms developing as a result of an inflammatory response to vaccination (6). Therefore, reactogenicity may influence vaccine acceptance. Although unpleasant, some degree of post-vaccination reactogenicity is inevitable and is expected to trigger a desirable vaccine immune response. BNT162b2 and ChAdOx1 nCoV-19 differ in their reactogenicity. Systemic reactogenicity following BNT162b2 vaccination is more common and severe after the second dose (3). By contrast, reactogenicity due to ChAdOx1 nCoV-19 is more frequent after the first dose than that after the second dose (5). Immune cells, such as mast cells and macrophages, are activated immediately after vaccination and release proinflammatory cytokines, such as interleukin (IL)-6 and tumor necrosis factor (TNF)-α (7–8). The post-vaccination kinetics of inflammatory cytokines differs for each vaccine platform and may be associated with reactogenicity (9). Therefore, it is imperative to determine the association of reactogenicity with immunogenicity and inflammatory response for the newly developed BNT162b2 and ChAdOx1 nCoV-19 vaccines. The association between reactogenicity and immunogenicity has been studied earlier; however, the results for various vaccines have been inconsistent (10–18). To clarify these inconsistencies, in this study, we monitored the antibody response between BNT162b2 and ChAdOx1 nCoV-19 up to 12 weeks post-vaccination, with respect to the kinetics of inflammatory cytokines and reactogenicity.

## Methods

### Study design

A total of 120 participants aged 19–59 years were enrolled at three teaching hospitals (Korea University Guro Hospital, Ajou University Hospital, and Kangnam Sacred Hallym University Hospital) between February 26 and March 12, 2021 (Clinical Trial Number: NCT05315856). This study was approved by the Institutional Review Board of each participating hospital, and written informed consent was obtained from all the study participants. With the vaccine shortage during COVID-19 pandemic, either BNT162b2 or ChAdOx1 nCoV-19 was assigned to each participant as per the Korean government policy, and the participants were not allowed to choose. Sixty participants were vaccinated with two doses of ChAdOx1 nCoV-19 (AstraZeneca) at an interval of 12 weeks, and the remaining 60 were immunized with two doses of the BNT162b2 (Pfizer-BioNTech) vaccine at an interval of 3 weeks. Participants with prior SARS-CoV-2 infection were excluded.

**Figure 1:**
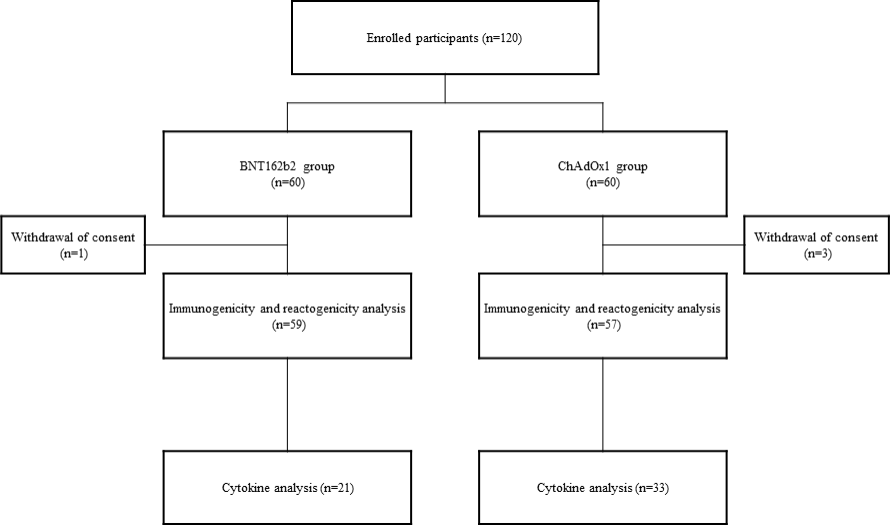
Diagram showing the study flow.

All participants were provided a self-administered questionnaire to collect data on adverse events following vaccination. For the serology study, blood samples were collected before vaccination, 3 weeks post-vaccination with the first and second doses, and 12 weeks after the second vaccination. Blood samples were obtained on day 3 after the first and second doses, and an additional informed consent for inflammatory cytokine analysis was obtained (**Figure 1**).

### Serologic assays

Immunoglobulin G (IgG) anti-S antibody levels were measured using the Elecsys^®^ Anti-SARS-CoV-2 S assay (Roche, Rotkreuz, Switzerland), according to the manufacturer’s protocol. A cutoff index (COI, signal sample/cutoff) of ≥ 1.0 was considered positive for anti-SARS-CoV-2 antibodies. Anti-N antibodies were measured in each participant using the SARS-CoV-2 IgG assay (Abbott Laboratories, Chicago, IL, USA) to exclude preceding SARS-CoV-2 infection.

For the neutralizing antibody (Nab) analysis, A plaque reduction neutralization test was performed using wild-type SARS-CoV-2 (hCoV/Korea/KCDC03/2020) and Delta variant (B.1.351 lineage, hCoV-19/Korea/KDCA55905/2021) to measure the level of Nabs against each strain (see supplementary appendix). A reduction in plaque count of 50% was calculated for the median neutralizing titer (ND50) using the Spearman–Karber formula and ND50 ≥ 1:20 was considered positive.

### Measurement of proinflammatory cytokines

Inflammatory cytokine levels were assessed immediately before and 3 d after the first and second vaccine doses. Freshly obtained samples were centrifuged, aliquoted, and stored at −80°C until further analysis. Serum levels of IL-6, TNF-α, and IL-1ß were measured using flexible customized bead-based multiplex panels for Luminex assays (Human Premixed Multi-Analyte Kit, R&D Systems Inc., Minneapolis, MN, USA). Cytokine concentrations were analyzed using the Luminex 100/200 system (Luminex, Austin, TX, USA), and the results were obtained using the MasterPlex software. Cytokine concentrations below the detection limit were assigned a value of zero.

### Adverse event assessment

We collected data on adverse events, including injection site erythema or swelling, fever, and muscle pain. Until 7 d post vaccination, the participants were requested to record the occurrence, severity, and duration of adverse events through a standardized electronic questionnaire. Local erythema or swelling was regarded positive if the affected area was larger than 2.5 cm in diameter. Fever was classified as grade 0 (<37.5 °C), grade 1 (from 37.5°C to 38.4°C) or grade 2 (>38.5°C). Systemic adverse events were graded as follows: grade 0, no systemic adverse event; grade 1, any adverse event that did not interfere with activity; and grade 2, any adverse event that interfered with daily activity. Furthermore, systemic adverse events were classified into two categories: (i) the highest severity of any adverse event reported by the participants, and (ii) with or without specific adverse events. Information on the use of antipyretics was collected after each vaccination.

### Statistical analyses

Continuous variables are presented as medians with interquartile ranges or means with standard deviations (SD), whereas categorical variables are presented as frequencies. The paired *t*-test or Mann–Whitney U test was used to compare continuous variables of paired data, and the Kruskal–Wallis test was used for continuous variables of multiple groups. The chi-square test or Fisher’s exact test was used for categorical variables. Repeated measures analysis of variance (ANOVA) was performed to analyze the time-dependent efficacy difference between the two vaccine groups. Spearman’s rank correlation was used to analyze the correlations, and multivariate linear regression analysis was used to adjust for confounding factors. Statistical significance was set at a two-sided *p*-value < 0.05.

## Results

### Characteristics of the study population

Four of the 120 participants requested withdrawal of consent for the follow-up study. A total of 59 participants in the BNT162b2 group and 57 participants in the ChAdOx1 nCoV-19 group were included in the immunogenicity analysis. Cytokine analysis was performed in 21 and 33 participants in the BNT162b2 and ChAdOx1 nCoV-19 groups, respectively, who consented to an additional visit 3 d after vaccination (**Figure 1**).

The mean age of the participants and the proportion of women in the BNT162b2 and ChAdOx1 nCoV-19 groups are listed in **Table 1**. The body mass index (BMI) between the two groups was comparable. All participants were healthy and did not have any underlying medical conditions. All participants showed negative anti-N antibodies at baseline, and none of the participants had SARS-CoV-2 infection during the study period.

**Table 1:** Patient demographics and adverse events after the first and second doses of BNT162b2 and ChAdOx1 nCoV-19.

	BNT162b2	ChAdOx1 nCoV-19	*p*-value
	n = 59	n = 57
Age (years), mean age ± SD	32.8 ± 7.5	34.0 ± 8.6	0.404
Female, n (%)	40 (67.8)	43 (75.4)	0.220
Body mass index	22.0 ± 3.4	22.4 ± 3.2	0.362
**Adverse events after the first dose**			
Local erythema/swelling, n (%)	3 (5.1)	8 (14.0)	0.122
Fever, n (%)			
Grade 0	49 (83.1)	10 (17.5)	<0.001
Grade 1	8 (13.6)	19 (33.3)	
Grade 2	2 (3.4)	28 (49.2)	
Muscle pain, n (%)			
Grade 0	17 (28.8)	8 (14.0)	0.069
Grade 1	31 (52.5)	30 (52.6)	
Grade 2	11 (18.6)	19 (33.3)	
Systemic event severity, n (%)			
Grade 0	16 (27.1)	2 (3.5)	<0.001
Grade 1	31 (52.5)	23 (40.4)	
Grade 2	12 (20.3)	32 (56.1)	
Antipyretic use, n (%)	17 (28.8)	49 (86.0)	<0.001
**Adverse events after the second dose**			
Local erythema/swelling, n (%)	2 (3.4)	5 (8.8)	0.268
Fever, n (%)			
Grade 0	30 (50.8)	52 (91.2)	<0.001
Grade 1	18 (30.5)	4 (7.0)	
Grade 2	11 (18.6)	1 (1.8)	
Muscle pain, n (%)			
Grade 0	8 (13.6)	24 (42.1)	<0.001
Grade 1	30 (50.8)	30 (52.6)	
Grade 2	21 (35.6)	3 (5.3)	
Systemic event severity, n (%)			
Grade 0	8 (13.6)	23 (40.4)	<0.001
Grade 1	29 (49.2)	31 (54.4)	
Grade 2	22 (37.3)	3 (5.3)	
Antipyretic use, n (%)	42 (71.2)	24 (42.1)	0.002

SD, standard deviation.

The adverse events after the first and second doses of the vaccinations are also summarized in **Table 1**. After each dose of vaccination, the prevalence of local adverse events (erythema or swelling) was similar in the BNT162b2 and ChAdOx1 nCoV-19 groups (after the first dose, *p* = 0.122; after the second dose, *p* = 0.268); however, fever and grade 2 systemic adverse events were significantly more frequent in the ChAdOx1 nCoV-19 group than in the BNT162b2 group after the first dose (*p* < 0.001). In comparison, the BNT162b2 group presented with fever, muscle pain, and any grade 2 systemic adverse event more frequently than the ChAdOx1 nCoV-19 group after the second dose (*p* < 0.001). Use of antipyretics was significantly more common in the ChAdOx1 nCoV-19 group than in the BNT162b2 group after the first dose (*p <*0.001); however, the reverse was true after the second dose (*p* = 0.002).

### Humoral immune response after vaccination

**Figure 2:**
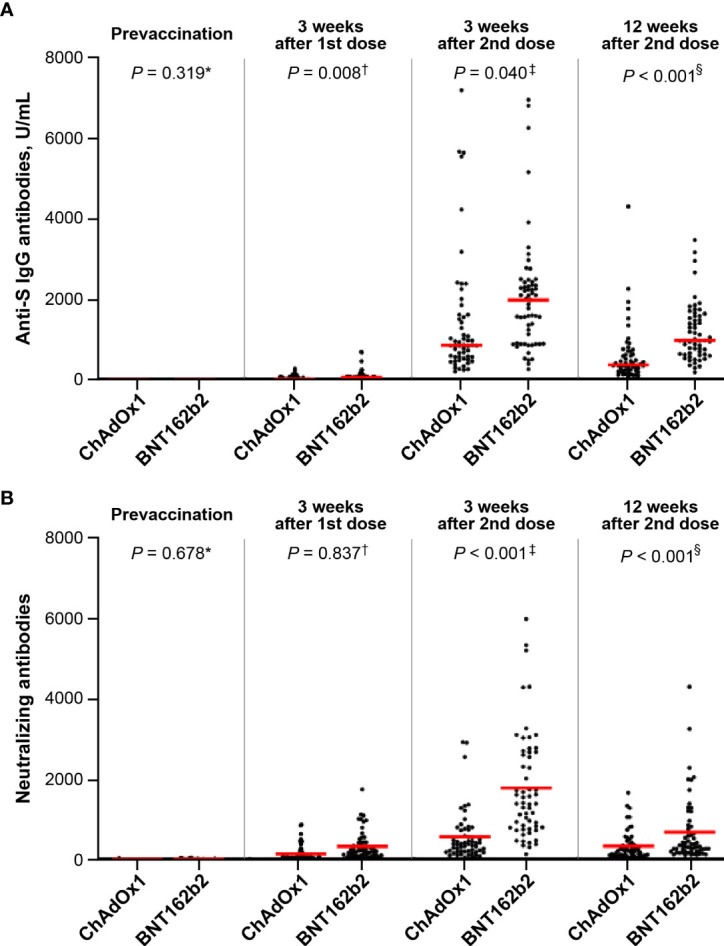
Comparison of antibody response between the ChAdOx1 nCoV-19 and BNT162b2 groups: **(A)** anti-S antibodies (mean ± standard deviation, U/mL): ^*^0 in ChAdOx1 and 0 in BNT162b2; ^†^50.0 ± 66.3 in ChAdOx1 and 101.5 ± 136.6 in BNT162b2; ^‡^1398.9 ± 1503.9 in ChAdOx1 and 2049.5 ± 1417.4 in BNT162b2; ^§^589.9 ± 679.7 in ChAdOx1 and 1171.2 ± 703.4 in BNT162b2 and **(B)** neutralizing antibodies (mean ± standard deviation): ^*^15.1 ± 37.1 in ChAdOx1 and 13.6 ± 10.8 in BNT162b2; ^†^293.2 ± 312.4 in ChAdOx1 and 325.7 ± 442.0 in BNT162b2; ^‡^642.5 ± 812.1 in ChAdOx1 and 1786.9 ± 1322.2 in BNT162b2; ^§^277.8 ± 301.9 in ChAdOx1 and 684.6 ± 806.7 in BNT162b2.

None of the participants tested positive for anti-S IgG antibodies before vaccination. The anti-S IgG antibody peaked after 3 weeks of the second dose (**Figure 2A**). During the study period, anti-S IgG antibody titer in the BNT162b2 group was approximately 2-fold higher than that in the ChAdOx1 nCoV-19 group at 3 weeks after the first dose (mean ± SD, 50.0 ± 66.3 U/mL *vs*. 101.5 ± 136.6 U/mL; *p* = 0.008), at 3 weeks after the second dose (mean ± SD, 1398.9 ± 1503.9 U/mL *vs*. 2049.5 ± 1417.4 U/mL; *p* = 0.040), and at 12 weeks after the second dose (mean ± SD, 589.9 ± 679.7 U/mL *vs*. 1171.2 ± 703.4 U/mL; *p* < 0.001).

As for the NAbs, the GMTs of ND50 against wild-type SARS-CoV-2 were 15.1 ± 37.1 in ChAdOx1 nCoV-19 group and 13.6 ± 10.8 in BNT162b2 group at baseline, respectively. After the first dose, the Nab response was similar between the ChAdOx1 nCoV-19 and BNT162b2 groups (mean ± SD, 293.2 ± 312.4 *vs*. 325.7 ± 442.0; *p* = 0.837) (**Figure 2B**). However, the Nab titer of the BNT162b2 group 3 weeks after the second dose was approximately 2.8-fold higher than that of the ChAdOx1 nCoV-19 group (mean ± SD, 1786.9 ± 1322.2 *vs*. 642.5 ± 812.1; *p* < 0.001). In both groups, the Nab titer peaked at 3 weeks after the second dose (BNT162b2 group *vs*. ChAdOx1 nCoV-19 group; mean ± SD, 684.6 ± 806.7 *vs*. 277.8 ± 301.9; *p* < 0.001), and decreased gradually until 12 weeks after the second dose. Repeated measures ANOVA revealed that the BNT162b2 group maintained higher Nab titers than the ChAdOx1 nCoV-19 group during the entire period (*p* < 0.001).

**Figure 3:**
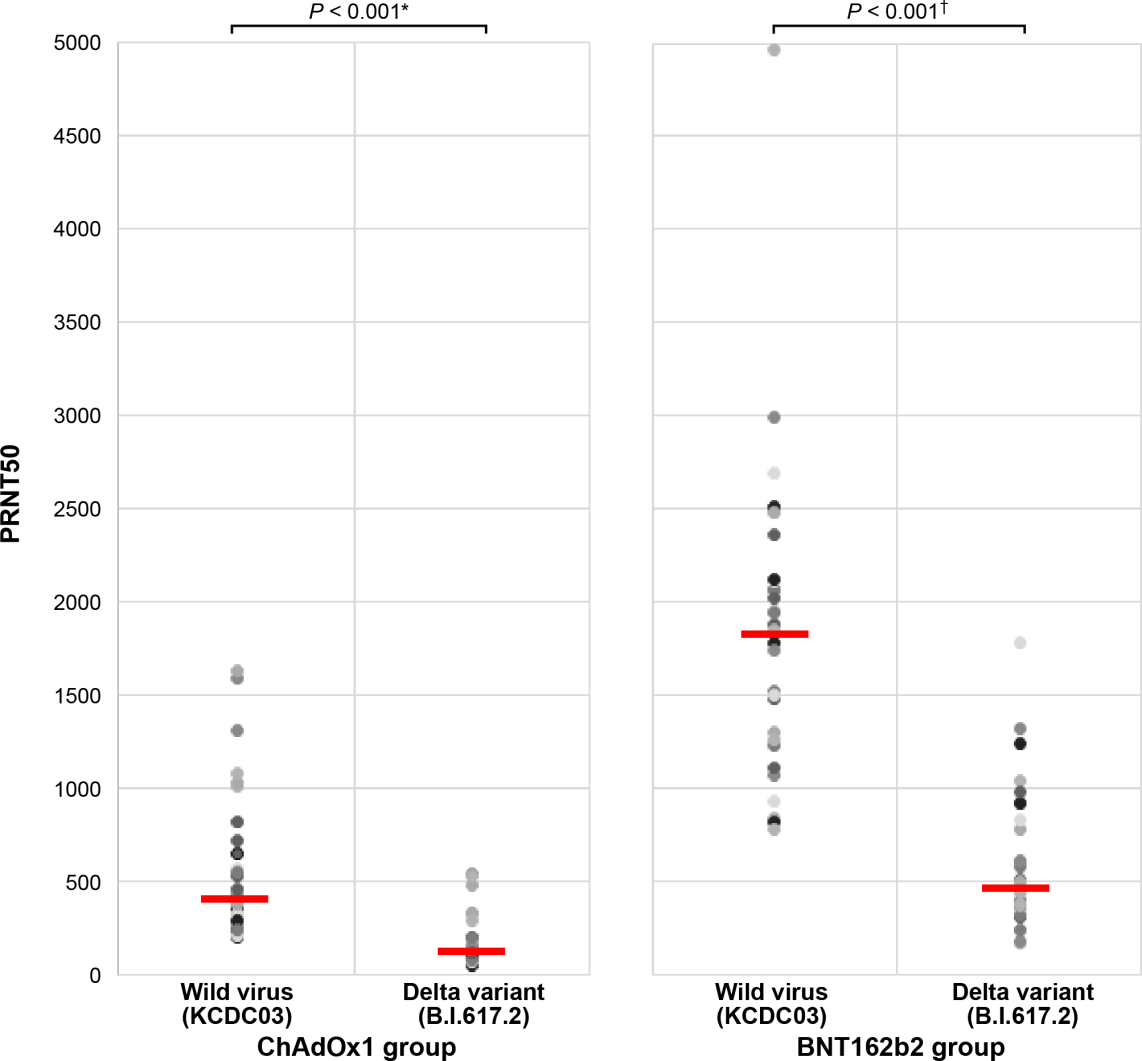
Comparison of antibody response against the wild-type and Delta variant strains between the ChAdOx1 nCoV-19 and BNT162b2 groups. *ChAdOx1 group (mean ± standard deviation): 480.1 ± 404.7 in wild-type virus and 148.3 ± 133.6 in Delta variant strain. †BNT162b2 group (mean ± standard deviation): 1706.1 ± 833.4 in wild-type virus and 487.4 ± 381.9 in Delta variant strain.

To evaluate the cross-reactive neutralizing activity, Nab responses to the wild-type SARS-CoV-2 and Delta variant strains were estimated in 30 randomly selected participants 3 weeks after the second dose. The Nab titer against the wild-type strain was significantly lower than those against the Delta variant strain in both the BNT162b2 (*p <*0.001) and ChAdOx1 nCoV-19 (*p <*0.001) groups (**Figure 3**). The BNT162b2 group had approximately 3-fold higher Nab titer against the wild-type and Delta variants than the ChAdOx1 nCoV-19 group (geometric mean titer: 480 *vs*. 1706 for wild-type strain, 148 *vs*. 500 for Delta variant strain; *p* < 0.001). Moreover, the reduction ratio of the ND50 titer between the Delta variant and the wild-type strain decreased by 3.43-fold and 3.68-fold in the ChAdOx1 nCoV-19 and BNT162b2 groups, respectively (*p* = 0.456).

### Kinetics of proinflammatory cytokines before and after COVID-19 vaccination

**Figure 4:**
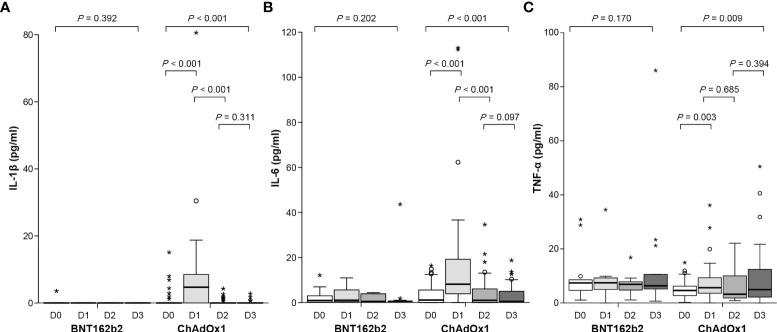
Concentration of proinflammatory cytokines before and after the first and second dose of ChAdOx1 nCoV-19 and BNT162b2: **(A)** IL-1β, **(B)** IL-6, and **(C)** TNF-α. Before the first dose (D0), day 3 after the first dose (D1), before the second dose (D2), and day 3 after the second dose (D3). Symbol * within graph is data value along with °.

On day 3 after the first dose of ChAdOx1 nCoV-19, the concentrations of proinflammatory cytokines IL-1β, IL-6, and TNF-α increased significantly to 4.61 (interquartile range [IQR]: 0–8.91) pg/mL (*p* < 0.001), 8.11 (IQR: 3.18–19.25) pg/mL (*p* < 0.001), and 4.39 (IQR: 2.77–7.61) pg/mL (*p* = 0.003), respectively, compared those before vaccination (0, 0.48 [IQR: 0–2.44] pg/mL, and 2.45 [IQR: 1.49–3.42] pg/mL, respectively) (**Figure 4** and **Supplementary Table 1**). However, the concentration of these cytokines before the second dose of ChAdOx1 nCoV-19 declined to pre-vaccination levels and did not increase on day 3 after the second dose. By contrast, the concentrations of proinflammatory cytokines did not show considerable changes after the first and second doses of BNT162b2. When evaluating the association between cytokine changes and systemic adverse reactions, the grade of any systemic adverse events in the ChAdOx1 nCoV-19 group was moderately correlated with the concentration of IL-6 (r = 0.54, *p* < 0.001) and modestly correlated with the concentration of IL-1β (r = 0.42, *p* < 0.001) (**Supplementary Figure 1**). By contrast, no significant association between the grade of systemic adverse events and the cytokine levels was observed in the BNT162b2 group.

### Association between reactogenicity and immunogenicity

**Figure 5:**
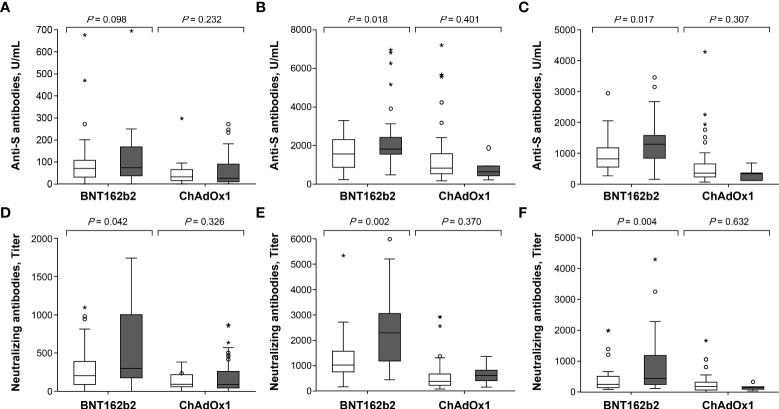
Comparison of antibody response after the first and second dose in ChAdOx1 nCoV-19 and BNT162b2 recipients with respect to febrile adverse events: **(A)** anti-S antibodies at 3 weeks after the first dose, **(B)** anti-S antibodies at 3 weeks after the second dose, **(C)** anti-S antibodies at 12 weeks after the second dose, **(D)** neutralizing antibodies at 3 weeks after the first dose, **(E)** neutralizing antibodies at 3 weeks after the second dose and **(F)** neutralizing antibodies at 12 weeks after the second dose. **□** Vaccine recipients without febrile adverse events. **▪** Vaccine recipients with febrile adverse events. Symbol * within graph is data value along with °.

In the BNT162b2 group, anti-S IgG and Nab titers were significantly higher in participants with temperature ≥37.5°C than in those without fever at 3 (*p* = 0.018 for anti-S IgG, *p* = 0.001 for Nab) and 12 weeks (*p* = 0.017 for anti-S IgG, *p* = 0.004 for Nab) after the second dose (**Figure 5** and **Supplementary Table 2**). The degree of fever was modestly correlated with anti-S IgG (r = 0.371, *p* = 0.004 at 3 weeks; r = 0.394, *p* = 0.002 at 12 weeks) and Nab titers (r = 0.397, *p* = 0.002 at 3 weeks; r = 0.404, *p* = 0.002 at 12 weeks) after the second dose of BNT162b2 (**Supplementary Table 3** and **Supplementary Figure 2**). However, anti-S IgG and Nab titers were not significantly associated with local erythema/swelling, myalgia, or any systemic adverse event (**Supplementary Figures 3–5**). In a multiple linear regression analysis model, anti-S IgG and Nab titers were significantly associated with the degree of fever following the second dose of BNT162b2, after adjustment for age, sex, BMI, and antipyretic use (*p* = 0.029 and *p* < 0.001, respectively) (**Supplementary Table 4**). By contrast, the ChAdOx1 nCoV-19 group did not show significant differences in anti-S IgG and Nab titers, based on the presence of local erythema or swelling and any systemic adverse events.

## Discussion

The results of this study indicated that post-vaccination immunogenicity in BNT162b2 recipients may be associated with febrile reaction, but not with local reactogenicity. In comparison, the systemic adverse events that followed the first dose of ChAdOx1 nCoV-19 may be associated with proinflammatory cytokine responses rather than with humoral immune responses. In addition, we directly compared humoral immunogenicity between BNT162b2 and ChAdOx1 nCoV-19 recipients. Anti-S IgG and Nab responses to the second BNT162b2 dose were significantly higher than those to the ChAdOx1 nCoV-19 vaccine. Although antibody titers decreased gradually over the 12-week period that followed the second dose, antibody titers in the BNT162b2 group remained at a higher level than those in the ChAdOx1 nCoV-19 group. Additionally, both vaccines showed a similar reduction ratio (~ 3.5) of neutralizing activity against the Delta variant versus wild-type SARS-CoV-2 at 3 weeks after the second dose. However, the BNT162b2 group showed a cross-reactive Nab titer that was 3-fold higher than that of the ChAdOx1 nCoV-19 group.

A significant difference was observed in the post-vaccination reactogenicity between BNT162b2 and ChAdOx1 nCoV-19. Systemic adverse events were more common in the ChAdOx1 nCoV-19 recipients after the first dose than after the second dose. By contrast, BNT162b2 recipients showed more frequent systemic adverse events after the second dose than after the first dose. The differences in reactogenicity could result from the different inflammatory and immune responses elicited by the two vaccines, representing two different vaccine platforms. In the ChAdOx1 nCoV-19 group, the grade of any systemic adverse event was moderately associated with the levels of proinflammatory cytokines, especially IL-6 and IL-1β, immediately after the first dose. However, BNT162b2 did not led to a significant increase in proinflammatory cytokine levels after both the first and second doses. Inflammatory response is essential for the activation of innate immunity and the induction of adaptive immunity following vaccination (8, 19). In particular, the proinflammatory cytokines IL-1β, IL-6, and TNF-α produced during the innate immune response provide signals for the differentiation of helper T-cells. Previous studies have reported that the inflammatory response to vaccination is substantially milder and more transient than those to natural infection (7, 20). By contrast, a hyperinflammatory response may cause immunotoxicity. Although the inflammatory response to vaccination is generally mild, some individuals might have a considerably elevated inflammatory response, which results in inflammation-related adverse events or the potential risk of developing immune-mediated disorders, such as Guillain-Barre syndrome (GBS) or immune thrombocytopenia. Adenovirus-vector vaccines, such as ChAdOx1 nCoV-19, are generally safe and are rarely associated with the risks of developing thrombosis with thrombocytopenia syndrome (TTS) and GBS (21). GBS is a well-known acute inflammatory disease of the peripheral nerves (22). TTS is caused by antibodies that recognize platelet factor 4 (PF4). Although the mechanisms triggering the production of anti-PF4 antibody are unclear, the generated antibodies may be implicated in a systemic inflammatory response wherein it complexes with the adenoviral hexon protein, a major coat protein binding to PF4, and stimulates proinflammatory milieu (23–24). Individuals with certain underlying conditions display amplified and prolonged inflammatory responses post-vaccination (25–26). Compared to BNT162b2, ChAdOx1 nCoV-19 may elicit a greater inflammatory response, which may cause potential immune-mediated disorders, such as TTS and GBS that are associated with adenovirus vector-based platforms (21, 27, 28).

Among the local and systemic adverse events following vaccination, antibody responses elicited by BNT162b2 vaccination immediately after the second dose appeared to be closely associated with febrile reactions, but not with local reactogenicity. The febrile response is believed to be mediated by the release of pyrogenic cytokines, such as TNF, IL-1, and IL-6 (29). Moreover, the antibody response following SARS-CoV-2 infection is higher in patients with severe disease than in those with mild disease (11, 30). Based on these findings, we postulate that higher immunogenicity after COVID-19 vaccination may be associated with greater reactogenicity. However, whether vaccine recipients with more severe adverse events are likely to exhibit enhanced vaccine-induced immune response, regardless of the vaccine platform, remains uncertain (10, 12–18). Additionally, the reactogenicity after BNT162b2 or ChAdOx1 nCoV-19 vaccination is only modestly associated with immunogenicity (10, 13, 16, 17). Various factors influence reactogenicity, including host characteristics, route of administration, and vaccine formulation (adjuvant, antigen type, and dose) (6). Although age, sex, and BMI did not affect antibody response in this study, ethnicity, psychological/physical stress inducers, and circadian cycles may have influenced reactogenicity and immunogenicity. This might justify why post-vaccination reactogenicity was modestly correlated with immunogenicity in this study.

After the 2-dose primary series vaccination, lower humoral immunogenicity was shown in ChAdOx1 nCoV-19 group compared to BNT162b2 group. In particular, unlike BNT162b2 group, a distinct immune boosting effect did not appear in ChAdOx1 nCoV-19 group after the second dose vaccination. Although antibody titers against chimpanzee adenovirus vector were not measured in this study, anti-vector antibodies induced during the first dose might have a chance to cause the immune interference during the second dose vaccination in ChAdOx1 nCoV-19 group. Actually, in the clinical trial of the COVID-19 vaccine using the human adenovirus serotype 5 (HAd5) vector, the immunogenicity was significantly lower when the pre-existing anti-HAd5 antibody titer was high (Nab titer ≥200) (31–32). When repeated vaccination is required, it is necessary to establish a vaccination schedule considering the half-life of the adenovirus vector.

This study has some limitations. First, blood samples for cytokine analysis were collected from less than 50% of the enrolled participants; hence, the conclusions based on these results should be made with caution. Second, we evaluated proinflammatory cytokines immediately before and 3 d after the first and second doses. However, the influenza vaccine is known to elicit a prominent inflammatory response 1–2 d post vaccination (7, 20). Since a time-lag can affect cytokine concentrations, further research is warranted to clarify the association between inflammatory and immune responses to vaccination.

In conclusion, prominent adverse events after the first dose of ChAdOx1 nCoV-19 were likely to be associated with an inflammatory response by cytokine production compared to that after a vaccine-induced immune response. By contrast, febrile adverse events after the second dose of BNT162b2 were positively correlated with vaccine-induced immune responses rather than inflammatory responses. We believe that the findings of the present study would be a milestone in understanding the association of reactogenicity with inflammatory response and immunogenicity after vaccination.

## Data availability statement

The original contributions presented in the study are included in the article/**Supplementary Material**. Further inquiries can be directed to the corresponding authors.

## Ethics statement

This study was reviewed and approved by Institutional Review Board of Korea University Guro Hospital. The patients/participants provided their written informed consent to participate in this study.

## Author contributions

JH, YS, and JS conceived and designed the study. JH, YS, and JS prepared the manuscript and analyzed the data. All authors contributed to the acquisition of the clinical and laboratory data. JH, YS, and JS contributed to data interpretation and statistical analysis. All the authors critically reviewed the manuscript for intellectual content and approved the final draft for submission.

## Funding

This work was supported by a grant from the Korea Disease Control and Prevention Agency [grant number: 2021-ER2603-01].

## Acknowledgments

We would like to thank all the participants who volunteered to participate in the study.

## Conflict of interest

The authors declare that the research was conducted in the absence of any commercial or financial relationships that could be construed as a potential conflict of interest.

## Publisher’s note

All claims expressed in this article are solely those of the authors and do not necessarily represent those of their affiliated organizations, or those of the publisher, the editors and the reviewers. Any product that may be evaluated in this article, or claim that may be made by its manufacturer, is not guaranteed or endorsed by the publisher.
