# Supplementary material for: COVID-19 vaccine type-dependent differences in immunogenicity and inflammatory response: BNT162b2 and ChAdOx1 nCoV-19
Source: Front Immunol. 2022 Sep 2;13:975363. doi: 10.3389/fimmu.2022.975363 (PMC9480614; doi:10.3389/fimmu.2022.975363)
Supplement: Supplementary file 1 [file DataSheet_1.docx]

Supplementary Material

# Supplementary Data

**Plaque reduction neutralization test**

A plaque reduction neutralization test (PRNT) was performed using wild-type SARS-CoV-2 (hCoV/Korea/KCDC03/2020) and Delta variant (B.1.351 lineage, hCoV-19/Korea/KDCA55905/2021) to measure the level of neutralizing antibodies (Nabs) against each strain. The mixture of serum dilution and virus (40 plaque forming units/well) was incubated at 37 °C for 2 h and added to the plate seeded with Vero E6 cells. Next, the plates were incubated at 37 °C for 1 h, followed by the addition of 0.5% agarose (Lonza, Basel, Switzerland). After incubation for 2-3 d, the cells were fixed with 4% paraformaldehyde and stained, to visualize the plaques. A 50% reduction in plaque count (PRNT50) was calculated for the median neutralizing titer (ND50) using the Spearman-Karber formula, and ND50 ≥ 1:20 was considered positive.

# Supplementary Tables and Figure

## Supplementary Table 1. Comparison of proinflammatory cytokines before and after the first and second dose of ChAdOx1 nCoV-19 and BNT162b2: (A) IL-1β, (B) IL-6, and (C) TNF-α. Before the first dose (D0), day 3 after the first dose (D1), before the second dose (D2), and day 3 after the second dose (D3)

|  | BNT162b2, median (IQR) | | | | P value | ChAdOx1 nCoV-19, median (IQR) | | | | P value |
| --- | --- | --- | --- | --- | --- | --- | --- | --- | --- | --- |
| pg/ml | D0 | D1 | D2 | D3 |  | D0 | D1 | D2 | D3 |  |
| IL-1β | 0 | 0 | 0 | 0 | 0.392 | 0 | 4.61 (0–8.91) | 0 | 0 | <0.001 |
| IL-6 | 0.13 (0–0.48) | 0.21 (0–2.11) | 0 (0–0.48) | 0 | 0.202 | 0.48 (0–2.44) | 8.11 (3.18–19.25) | 0.48 (0–3.12) | 0.48 (0–1.28) | <0.001 |
| TNF-α | 2.21 (1.25–3.42) | 3.31 (1.96–4.12) | 2.69 (1.94–3.10) | 3.41 (2.64–12.83) | 0.170 | 2.45 (1.49–3.42) | 4.39 (2.77–7.61) | 2.94 (1.49–10.75) | 4.93 (2.11–12.98) | 0.009 |

IQR, interquartile range

Supplementary Table 2. Comparison of post-vaccination antibody response (geometric mean titers) depending on the local and systemic adverse events after the first and second dose of BNT162b2 and ChAdOx1 nCoV-19

|  | BNT162b2 | | P value | ChAdOx1 nCoV-19 | | P value |
| --- | --- | --- | --- | --- | --- | --- |
|  | without adverse event | with adverse event |  | without adverse event | with adverse event |  |
| Anti-S IgG antibody titer depending on the local erythema/swelling, median (IQR) | |  |  |  |  |  |
| At 3 weeks after the first dose | 70.5  (31.9 - 113.0) | 13.9  (41.0 - NA) | 0.247 | 19.6  (7.3 - 62.8) | 49.6  (6.3 - 100.2) | 0.232 |
| At 3 weeks after the second dose | 1981.0  (895.0 - 2429.5) | 1858.0  (1360.0 - NA) | 0.916 | 853.0  (508.8 - 1617.0) | 865.0  (583.5 - 1072.0) | 0.967 |
| At 12 weeks after the second dose | 975.0  (637.0 - 1585.0) | 691.0  (594.0 - IV) | 0.318 | 370.0  (242.0 - 711.3) | 370.0  (279.0 - 527.5) | 0.967 |
| Anti-S IgG antibody titer depending on the fever, median (IQR) |  |  |  |  |  |  |
| At 3 weeks after the first dose | 70.7  (38.8 - 115.0) | 32.6  (12.2 - 72.9) | 0.098 | 14.2  (5.9 - 24.9) | 21.4  (7.3 - 76.6) | 0.232 |
| At 3 weeks after the second dose | 1568.0  (890.0 - 2305.8) | 2237.0  (1568.0 - 3051.5) | 0.018 | 857.5  (567.0 - 1604.3) | 667.0  (358.5 - 1608.0) | 0.401 |
| At 12 weeks after the second dose | 833.5  (559.5 - 1234.3) | 1306.0  (821.0 - 1792.0) | 0.017 | 379.5  (251.5 - 693.5) | 354.0  (139.5 - 542.0) | 0.370 |
| Anti-S IgG antibody titer depending on the myalgia, median (IQR) |  |  |  |  |  |  |
| At 3 weeks after the first dose | 73.8  (53.4 - 125.5) | 56.8  (21.6 - 101.0) | 0.150 | 23.0  (7.0 - 90.3) | 19.6  (7.3 - 62.8) | 0.884 |
| At 3 weeks after the second dose | 1256.5  (893.5 - 2342.3) | 2017.0  (1139.0 - 2434.0) | 0.458 | 748.5  (405.8 - 1410.3) | 958.0  (626.0 - 1737.5) | 0.106 |
| At 12 weeks after the second dose | 1004.0  (662.0 - 1488.5) | 975.0  (632.0 - 1581.0) | 1.000 | 363.0  (154.5 - 711.0) | 384.0  (286.0 - 664.5) | 0.447 |
| Anti-S IgG antibody titer depending on the antipyretic use, median (IQR) |  |  |  |  |  |  |
| At 3 weeks after the first dose | 68.4  (35.4 - 156.5) | 65.5  (19.0 - 76.4) | 0.209 | 20.5  (3.1 - 24.6) | 20.6  (7.3 - 75.7) | 0.371 |
| At 3 weeks after the second dose | 898.0  (858.0 - 2362.5) | 2101.0  (1383.3 - 2517.0) | 0.040 | 852.0  (491.5 - 1273.0) | 943.0  (574.0 - 2097.8) | 0.305 |
| At 12 weeks after the second dose | 759.0  (555.5 - 1388.0) | 1036.5  (749.3 - 1596.8) | 0.155 | 339.0  (199.5 - 607.5) | 427.5  (340.8- 761.8) | 0.085 |
| Neutralizing antibody titer depending on the local erythema/swelling, median (IQR) | |  |  |  |  |  |
| At 3 weeks after the first dose | 208.8  (103.3 - 426.0) | 70.9  (23.0 - IV) | 0.150 | 62.4  (31.4 - 131.1) | 130.1  (48.1 - 463.8) | 0.087 |
| At 3 weeks after the second dose | 1388.3  (778.8 - 2636.0) | 2176.0  (1573.8 - IV) | 0.400 | 375.9  (192.5 - 694.5) | 466.1  (271.0 - 1147.9) | 0.313 |
| At 12 weeks after the second dose | 323.1  (201.2 - 795.3) | 621.9  (384.5 - IV) | 0.491 | 204.7  (90.1 - 326.3) | 140.6  (99.8 - 677.0) | 0.651 |
| Neutralizing antibody titer depending on the fever, median (IQR) |  |  |  |  |  |  |
| At 3 weeks after the first dose | 210.4  (103.5 - 473.9) | 157.4  (66.6 - 275.1) | 0.042 | 41.6  (23.8 - 104.3) | 67.1  (36.9 - 167.9) | 0.326 |
| At 3 weeks after the second dose | 1004.3  (729.7 - 1563.1) | 2312.2  (1143.3 - 3102.4) | 0.002 | 360.1  (195.5 - 687.5) | 589.4  (255.8 - 1068.6) | 0.370 |
| At 12 weeks after the second dose | 275.7  (166.1 - 533.4) | 462.1  (271.3 - 1293.5) | 0.004 | 210.0  (96.0 - 348.5) | 179.9  (92.5 - 264.3) | 0.632 |
| Neutralizing antibody titer depending on the myalgia, median (IQR) |  |  |  |  |  |  |
| At 3 weeks after the first dose | 312.6  (113.4 - 887.3) | 188.0  (87.9 - 316.8) | 0.082 | 68.5  (32.4 - 256.7) | 63.4  (35.7 - 147.3) | 0.778 |
| At 3 weeks after the second dose | 1338.2  (770.3 - 2374.3) | 1548.0  (793.4 - 2671.8) | 0.588 | 391.1  (211.9 - 535.2) | 368.2  (179.8 - 722.9) | 0.910 |
| At 12 weeks after the second dose | 460.8  (176.6 - 677.3) | 340.5  (211.3 - 859.2) | 0.825 | 180.3  (91.4 - 468.9) | 209.3  (96.8 - 324.1) | 0.686 |
| Neutralizing antibody titer depending on the antipyretic use, median (IQR) |  |  |  |  |  |  |
| At 3 weeks after the first dose | 227.1  (95.0 - 527.7) | 179.9  (95.2 - 312.8) | 0.311 | 41.6  (26.2 - 72.5) | 67.1  (35.7 - 174.5) | 0.161 |
| At 3 weeks after the second dose | 1147.8  (739.7 - 1726.2) | 1591.2  (798.7 - 2697.9) | 0.192 | 313.8  (186.8 - 492.3) | 458.7  (212.1 - 976.8) | 0.070 |
| At 12 weeks after the second dose | 323.1  (167.4 - 671.2) | 372.0  (209.4 - 1024.0) | 0.493 | 180.7  (87.1 - 298.4) | 243.5  (104.0 - 567.7) | 0.096 |

IQR, interquartile range

**Supplementary Table 3.** Comparison of post-vaccination antibody response (geometric mean titers) by the grade of local and systemic adverse events after the first and second dose of BNT162b2 and ChAdOx1 nCoV-19

|  | BNT162b2 | | | P value | ChAdOx1 nCoV-19 | | | P value |
| --- | --- | --- | --- | --- | --- | --- | --- | --- |
|  | Grade 0 | Grade 1 | Grade 2 |  | Grade 0 | Grade 1 | Grade 2 |  |
| Anti-S IgG antibody titer depending on the fever, median (IQR) |  |  |  |  |  |  |  |  |
| At 3 weeks after the first dose | 70.7  (38.8 - 115.0) | 27.1  (11.7 - 69.9) | 137.1  (24.1 - NA) | 0.135 | 14.2  (5.9 -24.9) | 19.5  (3.3 - 74.8) | 22.0  (10.7 - 103.9) | 0.402 |
| At 3 weeks after the second dose | 1568.0  (890.0 - 2305.8) | 1878.0  (1153.8 - 2458.5) | 2263.0  (2101.0 - 3289.0) | 0.012 | 857.5  (567.0 - 1604.3) | 812.5  (353.5 - 1933.0) | 468.0  (NA) | 0.497 |
| At 12 weeks after the second dose | 853.5  (559.5 - 1234.3) | 974.0  (603.8 - 1596.8) | 1527.0  (1306.0 - 1910.0) | 0.004 | 379.5  (251.5 - 693.5) | 369.0  (194.3 - 621.0) | 138.0  (NA) | 0.352 |
| Neutralizing antibody titer depending on the fever, median (IQR) |  |  |  |  |  |  |  |  |
| At 3 weeks after the first dose | 210.4  (103.5 - 473.9) | 157.4  (58.1 - 296.4) | 174.5  (86.5 - NA) | 0.445 | 41.6  (23.8 - 104.3) | 63.4  (32.3 - 181.1) | 78.5  (38.5 - 165.9) | 0.330 |
| At 3 weeks after the second dose | 1004.3  (729.7 - 1563.1) | 2298.6  (1175.7 - 3142.9) | 2558.4  (1122.4 - 3100.8) | 0.004 | 360.1  (195.5 - 687.5) | 486.5  (191.8 - 747.7) | 1336.7  (NA) | 0.326 |
| At 12 weeks after the second dose | 275.7  (166.1 - 533.4) | 424.6  (267.0 - 1046.6) | 591.3  (318.3 - 2049.1) | 0.009 | 210.0  (96.0 - 348.5) | 190.0  (136.1 - 296.3) | 63.4  (NA) | 0.408 |
| Anti-S IgG antibody titer depending on any systemic adverse event, median (IQR) |  |  |  |  |  |  |  |  |
| At 3 weeks after the first dose | 76.0  (65.4 - 131.3) | 64.1  (28.8 - 98.0) | 41.5  (11.7 - 101.9) | 0.159 | 71.0  (6.0 - NA) | 19.5  (5.7 - 65.3) | 21.0  (7.3 - 86.0) | 0.870 |
| At 3 weeks after the second dose | 1256.5  (893.5 - 2342.3) | 1775.0  (875.0 - 2424.0) | 2101.0  (1568.0 - 2522.0) | 0.432 | 767.0  (465.0 - 1509.0) | 958.0  (585.0 - 1617.0) | 845.0  (468.0 - NA) | 0.481 |
| At 12 weeks after the second dose | 1004.0  (662.0 - 1488.5) | 946.0  (583.0 - 1612.5) | 1233.5  (655.5 - 1561.8) | 0.644 | 370.0  (177.0 - 749.0) | 384.0  (285.0 - 629.0) | 337.0  (138.0 - NA) | 0.805 |
| Neutralizing antibody titer depending on any systemic adverse event, median (IQR) |  |  |  |  |  |  |  |  |
| At 3 weeks after the first dose | 302.9  (112.5 - 932.9) | 183.8  (90.3 - 416.4) | 192.4  (80.2 - 304.5) | 0.290 | 148.5  (32.8 - NA) | 49.4  (25.0 - 140.0) | 73.9  (38.5 - 165.9) | 0.582 |
| At 3 weeks after the second dose | 1338.2  (770.3 - 2374.3) | 1548.0  (796.9 - 2435.3) | 1513.8  (701.8 - 2828.4) | 0.855 | 398.6  (209.1 - 552.1) | 352.0  (177.9 - 729.5) | 802.7  (561.5 - NA) | 0.137 |
| At 12 weeks after the second dose | 460.8  (176.6 - 677.3) | 311.5  (179.6 - 537.0) | 419.3  (218.1 - 1407.5) | 0.268 | 180.7  (88.3 - 502.9) | 209.3  (99.1 - 319.8) | 81.1  (63.4 - NA) | 0.663 |

IQR, interquartile range

**Supplementary table 4.** Multivariate analysis for the factors related to post-vaccination antibody responses after the first and second dose of BNT162b2

| variables | Anti S-IgG response  after first dose | |  | Neutralizing antibody response  after first dose | |  | Anti S-IgG response  after second dose | |  | Neutralizing antibody response  after second dose | |
| --- | --- | --- | --- | --- | --- | --- | --- | --- | --- | --- | --- |
|  | Standardized β | *p*-value |  | Standardized β | *p*-value |  | Standardized β | *p*-value |  | Standardized β | *p*-value |
| Age | -0.215 | 0.041 |  | -0.071 | 0.501 |  | 0.135 | 0.170 |  | -0.051 | 0.582 |
| Male | 0.017 | 0.892 |  | 0.02 | 0.875 |  | 0.770 | 0.509 |  | 0.02 | 0.859 |
| Body mass index | 0.122 | 0.323 |  | -0.032 | 0.799 |  | -0.088 | 0.452 |  | 0.065 | 0.558 |
| Local erythema/swelling | 0.008 | 0.934 |  | 0.017 | 0.862 |  | -0.117 | 0.206 |  | -0.024 | 0.779 |
| Fever | 0.032 | 0.852 |  | -0.011 | 0.947 |  | 0.241 | 0.029 |  | 0.444 | < 0.001 |
| Myalgia | 0.019 | 0.914 |  | 0.013 | 0.942 |  | 0.756 | 0.129 |  | 0.335 | 0.474 |
| Systemic adverse events | -0.075 | 0.740 |  | -0.186 | 0.415 |  | -0.800 | 0.120 |  | -0.341 | 0.482 |
| Antipyretic use | -0.222 | 0.077 |  | -0.129 | 0.306 |  | 0.268 | 0.013 |  | 0.150 | 0.136 |

## Supplementary Figures





**Supplementary figure 1.** Concentration of proinflammatory cytokines by the grade of systemic adverse events after the first and second dose of ChAdOx1 and BNT162b2: (A) IL-1β, (B) IL-6 and (C) TNF-α.

^*^Correlation coefficient (r_s_) = 0.423, *p* < 0.001, ^†^Correlation coefficient (r_s_) = 0.540, *p* < 0.001





**Supplementary figure 2.** Comparison of post-vaccination antibody response (geometric mean titers) by the grade of febrile events after the first and second dose of BNT162b2 and ChAdOx1: (A) anti-S antibodies at 3 weeks after the first dose, (B) anti-S antibodies at 3 weeks after the second dose, (C) anti-S antibodies at 12 weeks after the second dose, (D) neutralizing antibodies at 3 weeks after the first dose, (E) neutralizing antibodies at 3 weeks after the second dose and (F) neutralizing antibodies at 12 weeks after the second dose

**□** Grade 0 (G0): body temperature ≤ 37.4℃, **■** Grade 1 (G1): 37.5℃ ≤ body temperature ≤ 38.4℃, **■** Grade 2 (G2): body temperature ≥ 38.5℃

^*^Correlation coefficient (r_s_) = 0.371, *p* = 0.004, ^†^Correlation coefficient (r_s_) = 0.394, *p* = 0.002, ^‡^Correlation coefficient (r_s_) = 0.397, *p* = 0.002, ^§^Correlation coefficient (r_s_) = 0.404, *p* = 0.002





**Supplementary figure 3.** Comparison of antibody response (geometric mean titers) after the first and second dose in ChAdOx1 and BNT162b2 recipients with respect to the local adverse events: (A) anti-S antibodies at 3 weeks after the first dose, (B) anti-S antibodies at 3 weeks after the second dose, (C) anti-S antibodies at 12 weeks after the second dose, (D) neutralizing antibodies at 3 weeks after the first dose, (E) neutralizing antibodies at 3 weeks after the second dose and (F) neutralizing antibodies at 12 weeks after the second dose

**□** vaccine recipients without the local adverse events, **■** vaccine recipients with the local adverse events





**Supplementary figure 4.** Comparison of antibody response (geometric mean titers) after the first and second dose in ChAdOx1 and BNT162b2 recipients with respect to myalgia: (A) anti-S antibodies at 3 weeks after the first dose, (B) anti-S antibodies at 3 weeks after the second dose, (C) anti-S antibodies at 12 weeks after the second dose, (D) neutralizing antibodies at 3 weeks after the first dose, (E) neutralizing antibodies at 3 weeks after the second dose and (F) neutralizing antibodies at 12 weeks after the second dose.

**□** vaccine recipients without myalgia, **■** vaccine recipients with myalgia





**Supplementary figure 5.** Comparison of antibody response (geometric mean titers) after the first and second dose in ChAdOx1 and BNT162b2 recipients by the grade of any systemic adverse events: (A) anti-S antibodies at 3 weeks after the first dose, (B) anti-S antibodies at 3 weeks after the second dose, (C) anti-S antibodies at 12 weeks after the second dose, (E) neutralizing antibodies at 3 weeks after the first dose, (F) neutralizing antibodies at 3 weeks after the second dose and (G) neutralizing antibodies at 12 weeks after the second dose.

**■** Grade 1 (G1): any systemic adverse events that did not interfere with activity, **■** Grade 2 (G2): any systemic adverse events that interfered with daily activity
